# Provider-recipient perspectives on how social support and social identities influence adaptation to psychological stress in sport

**DOI:** 10.3389/fpsyg.2022.940747

**Published:** 2022-08-10

**Authors:** Chris Hartley, Pete Coffee, Purva Abhyankar

**Affiliations:** ^1^Faculty of Health Sciences and Sport, University of Stirling, Stirling, United Kingdom; ^2^School of Social Sciences, Heriot-Watt University, Edinburgh, United Kingdom; ^3^Faculty of Natural Sciences, University of Stirling, Stirling, United Kingdom

**Keywords:** stress, social support, social identity, qualitative, thematic analysis, naturalistic paradigm, rugby, academy sport

## Abstract

Psychological stress can be both a help and a hindrance to wellbeing and performance in sport. The provision *and* receipt of social support is a key resource for managing adaptations to stress. However, extant literature in this area is largely limited to the *recipient’*s perspective of social support. Furthermore, social support is not always effective, with evidence suggesting it can contribute to positive, negative, and indifferent adaptations to stress. As such, we do not know *how* social support influences adaptations to stress in sport. The social identity approach may explain how social support can lead to both positive and negative adaptations to stress. Our purpose in this study was to explore how social support and social identities influence adaptations to stress in a Rugby Academy Programme. Using qualitative methods within a naturalistic research paradigm, semi-structured interviews were conducted with Rugby Academy co-ordinators (*n* = 6) and players (*n* = 12), and four focus groups were conducted with teams of support staff (*n* = 18). Data were analyzed using reflexive thematic analysis, which generated seven sub-themes categorized into two higher-order analytical themes. Our results demonstrate that group-based perceptions of social support influence adaptations to stress. Specifically, whether social support influences positive, negative, or indifferent adaptations to stress depended on (1) social factors influencing the nature of social support, and (2) social factors influencing the provision and receipt of social support. These findings advance our understanding of how adaptations to stress are influenced by social support. Implications are offered for how organizations, teams, and practitioners can facilitate positive adaptations to stress in sport.

## Introduction

It is established that psychological stress can be both a help and a hindrance to wellbeing and performance in sport (Sarkar et al., 2015). In this regard, social support is considered a key resource for managing adaptations to stress, with evidence for positive, negative, and indifferent adaptations (Sheridan et al., 2014). These adaptations, in turn, affect a range of outcomes such as performance, mental health, and physical health (Cohen et al., 2000; Freeman et al., 2014; Rees, 2016; Hartley and Coffee, 2019). However, there is a lack of evidence informing *how* social support influences these positive, negative, and indifferent adaptations to stress. Gaining a better understanding of this may inform how organizations, teams, and practitioners can support positive adaptations to stress—and more generally inform how these groups can understand, safeguard, and support wellbeing and performance in sport (e.g., Henriksen et al., 2020; Sly et al., 2020).

The sport environment involves exposure to a range of stressors (e.g., competitive performance, organizational and non-sporting stressors; Sarkar and Fletcher, 2014). This may result in the experience of stress (Gustafsson et al., 2008)—a state whereby athletes anticipate personally relevant events based on their appraisal of situational demands and available resources (e.g., to cope; Lazarus and Folkman, 1984; Meijen et al., 2020). Stress can negatively impact outcomes in sport, including reducing psychological wellbeing (Malinauskas and Malinauskiene, 2018) and increasing burnout amongst athletes (Gustafsson et al., 2017). However, exposure to stressors does not necessarily lead to negative outcomes. Depending on one’s adaptation to stress (i.e., the outcome of the appraisal process as based on one’s physiological changes, predispositions, and cognitive appraisals that mark challenge and threat states; see Meijen et al., 2020), some individuals may withstand and even experience positive outcomes in the presence of stressors (Galli and Gonzalez, 2015; Bryan et al., 2019). For example, positive adaptations to stress may result in athletes experiencing stress-related growth and improved mental toughness (Park et al., 1996; Tedeschi and Calhoun, 1996; Bell et al., 2013). There are a range of factors that influence whether positive, negative, or indifferent adaptations to stress are likely to happen (Fletcher and Sarkar, 2013; Meijen et al., 2020). In this regard, social support is considered a key resource for influencing adaptations to stress (Hartley and Coffee, in press; Sarkar and Hilton, 2020).

Social support is a complex and multifaceted construct, encompassing both structural (i.e., number and type of relationships) and functional components (i.e., particular functions and purposes) of interpersonal relationships (Cohen et al., 2000; Lakey, 2010). This includes both socially supportive relationships and actions, often involving the (actual or perceived) exchange or availability of resources intended to benefit the recipient (Bianco and Eklund, 2001). Researchers (e.g., Marigold et al., 2014; Coussens et al., 2015) have demonstrated that recipients differ in their perceptions of perceived and received support (this may also vary if assessed at the team vs. individual level; Coffee et al., 2017). As such, it has been suggested that researchers broaden their investigations to include the provision of *enacted* support (i.e., the manifest support actions from providers of support; Barrera, 1986), as well as the *receipt* of social support (perceived and received). Indeed, most of the extant literature documents the experiences of *recipients* of social support (e.g., by investigating perceptions of support that are available if needed and/or received from others; Freeman and Rees, 2010; Hartley and Coffee, 2019). Yet the experiences of *providers* of social support remain under-investigated. Capturing both perspectives of providers (enacted) and recipients (perceived, received) could allow researchers to better understand *how* social support influences adaptations to stress.

In both the sport and broader psychology literature, there is evidence to suggest that while social support may allow for beneficial adaptations to stress, it may also result in indifferent and even negative adaptations to stress (Arnold et al., 2018; Hartley and Coffee, in press). For example, poorly delivered social support may lack clear understanding of a recipient’s needs, draw attention to a recipient’s incompetence, undermine goal-pursuits, and even increase perceptions of pressure, risk of burnout, and dropout rates amongst athletes (e.g., Gleason et al., 2008; Sheridan et al., 2014; Gouttebarge et al., 2015; Prinz et al., 2016; Hartley and Coffee, 2019). Indeed, research into the social factors surrounding athlete mental health and dual career programs highlights a paradox. On one hand, a range of formal and informal sources of support may be provided to help facilitate positive mental health and career transitions for athletes. On the other hand, athletes may experience stress and stigma when accessing such support due to concerns over it being perceived by others in their team as a sign of “weakness” and fearing deselection (Brown et al., 2018; Butler et al., 2018). This places the study of social support in sport at a juxtaposition—where it can be both the key to coping with and achieving positive adaptations to stress or the source of negative adaptations to stress (Hartley et al., 2020). As such, it is currently unclear *how* social support influences adaptations to stress, and how exactly organizations, teams, and practitioners can facilitate positive adaptations to stress in sport.

Two predominant approaches in the sport literature help to explain how social support influences adaptations to stress. First, according to transactional theories of stress (Cox, 1978; Lazarus and Folkman, 1984; Lazarus, 1999), perceived and received support are thought to influence adaptations to stress by making unique contributions to the causal chain from stressor to outcome (Cohen and Wills, 1985). Specifically, perceived support is theorized to influence perceived capabilities and resources to cope with stressors (i.e., affecting both primary and secondary stress-appraisals; Freeman and Rees, 2009), whereas received support is theorized to intervene as a coping resource once the sensation of stress is experienced (i.e., affecting secondary stress-appraisal and buffering the effects of stress; Cohen et al., 2000; Bianco and Eklund, 2001). Second, the revised theory of challenge and threat states in athletes (Meijen et al., 2020) posits that the social environment is inherent during stress reappraisal. Specifically, this emphasizes the degree to which resultant challenge and threat states are influenced by the iterative reappraisal of situational demands vs. available coping resources (i.e., a threatening stressor may not necessarily result in poor performance due to the perception of available support).

While the above approaches have furthered understanding of how social support influences adaptations to stress, they are limited in their account of the social nature of social support. In reference to existing approaches, Hartley et al. (2020) make the following three points. First, existing approaches conceptualize the experience of social support in *personal* terms (e.g., focus on the recipient only, “*Me*”). However, as already argued above, social support involves an exchange between (potentially multiple) providers and recipients. As such, salient group dynamics will contribute to social support’s influence on adaptations to stress. Second, while existing approaches emphasize the role of the social environment during stress re/appraisal (e.g., perceived social support; Meijen et al., 2020), they do not offer theoretical explanations for how *social factors* within these environments influence the effects of social support. Notably, social factors such as group memberships (“*We*”) and their associated identity content (i.e., values underpinning a group such as win at all costs vs. fair play) are likely to influence group members’ experience of stress and social support. Specifically, the characterizing identity of a group may influence what stressors are considered most pertinent to group members’ needs, as well as their trust and engagement with certain support providers (Nicholson et al., 2011). Third, existing approaches fail to explain why social support can result in negative adaptations as discussed above. In this regard, examining the social nature of social support (e.g., how group norms influence what types of support are considered acceptable vs. inacceptable for group members) could explain why well-intentioned and optimally matched social support may result in negative adaptations to stress.

To address the above limitations and more fully understand *how* social support influences adaptations to stress, researchers have suggested turning to the social identity approach (social identity theory and self-categorisation theory; Hartley et al., 2020). Social identity theory (Tajfel, 1972; Turner, 1982) posits that a person’s sense of self is based on their group membership(s), and that these group memberships are an important source of pride and self-esteem (Tajfel and Turner, 1979). In an extension of this, self-categorisation theory (Turner et al., 1987, 1994) explains that self-categorizing oneself as a group member (i.e., depersonalizing and adopting a social identity of “*Us*”) influences how the self is defined (i.e., in group-like social terms). Researchers from other areas of psychology have demonstrated how these social identity and self-categorisation processes underpin the experience of social support (e.g., Haslam et al., 2012, 2018; Slater et al., 2013). For example, shared social identities provide the basis for mutual influence by motivating group members to achieve agreement and co-ordination over their social support behaviors (Haslam et al., 1998; Rees et al., 2015). Furthermore, ingroup-outgroup boundaries may discriminately influence how support is provided and engaged with (e.g., by withholding support from outgroup members, and favorably engaging with ingroup support providers; Tajfel and Turner, 1979; Nicholson et al., 2011).

In this regard, the social identity approach may help address some of the limitations of extant approaches to the study of social support in sport (see, Hartley et al., 2020). First, the approach provides a theoretical framework to conceptualize and examine social support in *social* terms by acknowledging that it involves the provision (enactment) *and* receipt of (perceived/received) social support within groups (e.g., as seen in multidisciplinary support teams and sport organizations; Reid et al., 2004). Second, the approach can help explain how *social factors* might influence the effects of social support, due to postulating how the nature of shared group memberships condition the provision and receipt of social support (e.g., support provided by a rival team member may not have the same meaning or impact as support provided by a member of one’s own team; Foddy et al., 2009). Third, the approach might explain *how*—depending on whether social support is seen to align or conflict with a group’s identity-based norms—social support results in positive, negative, and indifferent adaptations to stress. For example, receiving support for a mental health need may conflict with a group identity centered on “performance” and therefore result in negative adaptations to stress (e.g., due to raising concern over deselection; Wood et al., 2017; Brown et al., 2018). Conversely, receiving support for a mental health need may align with a group identity centered on “welfare and wellbeing” and therefore result in positive adaptations to stress (e.g., due to affirming the group’s espoused identity).

Despite theoretical potential for the social identity approach to address the extant limitations on this topic, it is yet to be investigated. As such, our purpose in this study was to explore how social support and social identities influence adaptations to stress in a Rugby Academy Programme. Doing so may better inform *how* social support influences positive, negative, and indifferent adaptations to stress, and therefore provide implications for how organizations, teams, and practitioners can support positive adaptations to stress. Building on the literature above, we formulated three research questions (RQs):

RQ1: How can we understand social support using the social identity approach?

RQ2: How can social factors influence the effects of social support?

RQ3: How can we explain social support’s positive, negative, and indifferent adaptations to stress in sport?

## Materials and methods

### Design

A naturalistic research paradigm with qualitative methods was adopted to generate comprehensive and ecologically situated insights (Guba and Lincoln, 2004). This approach acknowledges that differences may be noted across multiple perspectives of the social support experience (e.g., Marigold et al., 2014; Coussens et al., 2015), that social support is contextually bound, and that researchers are unlikely to be completely detached from their line of inquiry. Qualitative methods also help capture the understudied perspectives of both providers and recipients of support, as well as the nuanced influence of social support and social identities on adaptations to stress.

#### Ontological and epistemological paradigm

Our underpinning philosophical orientation to this study was based on a critical realist perspective. Specifically, our ontological position was that a hard, mind-independent reality exists (ontological realism). Our epistemological position was that, although a mind-independent reality exists, it is impossible to reach an absolute understanding of that reality as all methods of enquiry rely on participants’ and researchers’ interpretations of that reality. As such, we could only work toward a closer understanding of reality by adopting a *critical* epistemological position of realism (Atkinson, 2012). This philosophical orientation was reflected in the present study’s research questions, where the influence of social support and social identity on adaptations to stress were regarded as objective phenomena, while acknowledging these would be influenced by the meanings and interpretations that participants give to their experiences of providing and receiving social support. The critical realist orientation was also reflected in our methods of data collection and analysis, where our role as researchers were recognized from the outset (Haegele and Hodge, 2015). Specifically, the lead researcher (CH) was recognized as being a white male sport psychologist with doctoral-level knowledge of the phenomena of interest, who had personal experience of competing and working in non-elite/elite sport. Through the process of reflective journaling and supervision, the lead researcher initially became aware of the potential for their lived experience to result in subjective biases that might impact on the study (e.g., by interpreting participants’ experiences of group life and social support with reference to the researcher’s own personal experiences, or interpreting data to align with pre-existing social support or social identity theory). The impact of these subjective biases on the study was managed by employing processes that support methodological quality and rigor as detailed below.

#### Methodological quality and rigor

Quality and rigor of the findings were supported through several processes. First, the study used established methods for data collection and analysis. Second, transferability of the findings was supported by providing rich and accessible description with supporting quotations directly from the study participants (Ungar, 2003) and by following the Consolidated Criteria for Reporting Qualitative Research (Tong et al., 2007) to report important aspects of the research process throughout. Third, the role of the lead researcher and their subjective biases were managed throughout data collection and analysis by maintaining a reflective diary, as well as using the second and third authors (researchers with doctoral-level knowledge and publications in the social psychology of sport and health) as critical friends to acknowledge and challenge lived experience (Smith and McGannon, 2018). For example, after every interview and focus group the lead researcher wrote a structured reflection using Gibbs’ (1988) cycle to evaluate and analyze the encounter, followed by discussing these reflections with the second and third authors (this benefitted the data collection process by, for example, helping the lead researcher to avoid interpreting participants’ responses with reference to their own personal experience). Fourth, the second and third authors were also used as critical friends to evaluate the credibility of the analysis and critically challenge the interpretations being generated. This was done by reviewing transcripts together, having regular discussions about themes and asking questions about their relationships to pre-existing theory, and actively looking for contrary explanations (Ronkainen and Wiltshire, 2021). These processes resulted in several revisions to the content and interpretation of the themes and sub-themes to ensure they were distinct and relevant to the research questions.

### Participants and sampling

This study received ethical approval from the University of Stirling and was conducted as part of a research project investigating the provision and receipt of a holistic support program in a Rugby academy (termed Academy) spread across regional training centers. As such, our sample consisted of three different Academy sub-populations: (1) support program co-ordinators; (2) teams of multidisciplinary support staff, and (3) players. Participants were only recruited if they were over 16 years of age and able to provide voluntary informed consent on the basis of having a full understanding of the research project and engagement expectations (participants under the age of 18 also required parental consent and the presence of a responsible adult during data collection). To ensure a broad range of experiences and views, a cross-section from the Academy (*N* = 36) was gathered using purposive sampling bespoke to each sub-population (Ritchie et al., 2003), as follows:

#### Academy co-ordinators

Participant suitability was determined through collaborating with the National Governing Body’s (NGB) human resources team. Based on the study research questions, the inclusion criteria were to be involved in the NGB’s procurement and co-ordination of performance and welfare support for staff and players, and to have engagement with the Academy support program. To maximize variation in perspectives, co-ordinators were purposively sampled to comprise males and females from different age and staff groups. A gender-representative total of six co-ordinators consisting of directorial- and managerial-level staff from performance, human resources, and medical teams were identified, approached, and successfully recruited *via* email invitation (35–54 years of age; 2 females).

#### Academy support staff

Participant suitability was determined through collaborating with the NGB’s human resources team and regional Academy managers. Based on the research questions, the inclusion criterion was to be working as part of a multidisciplinary team of Academy support staff and therefore engaged with the Academy support program. To ensure diverse and representative views, 20 eligible male and female members of support staff from varied professional disciplines (rugby coaches, strength and conditioning (S&C) coaches, physiotherapists, and Academy managers; *n* of smallest subgroup = 4) were identified and approached *via* email invitation. There were two eligible male S&C coaches who responded to the invite for study participation but were unable to participate on the day of data collection. As such, a gender-representative total of 18 participants were recruited (27–55 years of age; 2 female), incorporating support staff from all regional training centers.

#### Academy players

Participant suitability was determined through collaborating with Academy managers. Based on the research questions, the inclusion criterion was to be a current Academy player and therefore engaged with the Academy support program. To maximize variation in perspectives, 13 eligible male and female Academy players were identified and approached *via* email invitation. One eligible participant did not respond to the invite for study participation. As such, a gender-representative total of 12 participants were recruited (17–26 years of age; 3 female), incorporating players from all regional training centers.

### Data collection

The Academy sub-populations warranted different methods for eliciting data. Face-to-face semi-structured interviews were used to investigate social support amongst Academy co-ordinators and Academy players as this method is well suited for gathering individual accounts. However, it is common in sport settings for multidisciplinary support to be provided by *teams* of staff (Reid et al., 2004; Fletcher and Wagstaff, 2009), hence individual interviews were deemed unsuitable for this sub-population. Instead, focus groups were used for eliciting data from Academy support staff, as this allowed for the exploration of shared perspectives and understandings amongst these teams (thereby allowing the group to become more than “the sum of its parts”; Krueger and Casey, 2009).

#### Semi-structured interviews and focus group schedules

Considering that the Academy sub-populations warranted different methods for eliciting data, a semi-structured approach was used for interviews and focus groups given its flexibility in questioning (Robson, 2002). This allowed individual questions to be phrased differently depending on the individual or focus group being interviewed, while keeping the discussion centered around the research objectives. Based on the study research questions and extant literature on social support and social identity in sport, we constructed an interview schedule containing four sections on background information, social support, stressors and support needs, and social identities. Prior to data collection, the lead researcher undertook four pilot interviews (not included in dataset) to check and revise the interview questions and technique. For example, piloting resulted in the reordering and rewording of some questions to improve understandability (e.g., asking participants about their group memberships rather than their social identities). The final schedule began by asking participants about their professional background, followed by questions about their understanding of social support in sport (e.g., what types of support are provided and received within the Academy, and the resultant effects of this social support). Participants were then asked about the stressors and support needs of players, how these were identified, and how the support addressed them. Finally, participants were asked to describe their group membership/s with the Academy (e.g., if they had common group memberships with others in the Academy, what these group memberships meant to them), and to describe if/how these group memberships influenced their behavior (specifically, the provision and receipt of social support).

#### Procedure

Face-to-face interviews and focus groups were conducted in locations convenient to participants. Prior to data collection (and again on the day of data collection), participants were provided with study information (i.e., study purpose, procedure, use of audio-recorded data) and consent sheets. There were no incentives for study participation. Interviews and focus groups were conducted by the first author who was familiar with qualitative interviewing. Academy co-ordinator and player interviews lasted between 22 and 56 min (*M* = 32.55, *SD* = 8.91), while support staff focus groups lasted between 41 and 64 min (*M* = 54.55, *SD* = 9.36). The first author then transcribed all audio recordings verbatim (resulting in 236 pages of transcript), which also served as part of the familiarization stage for subsequent analyses. Following transcription and initial analyses, participants were provided with their transcript-copies and preliminary results for the purposes of member reflections (i.e., to seek their views on our interpretations of their data with the awareness that it may result in competing alternative explanations; Smith and McGannon, 2018; Ronkainen and Wiltshire, 2021). No participants provided comments or requested any changes.

### Analyses

Reflexive thematic analysis was chosen given its flexible approach to providing both descriptive and interpretative accounts of data (Braun and Clarke, 2006, 2019; Braun et al., 2016). The analysis was conducted in six stages, initially using an inductive approach to allow for the generation of diverse and novel themes, followed by a deductive approach to allow for themes to be linked to pre-existing theory (Ritchie and Spencer, 2002). In order to evaluate and modify themes, the first author maintained a reflective diary and engaged in disputative conversations with the second and third authors as critical friends throughout the process of analysis to consider competing alternative explanations (Ronkainen and Wiltshire, 2021). This also supported the use of an inductive and deductive approach to critical realism—by allowing new insights to be generated from the data while being aware of and challenging the impact of the first author’s subjective biases and lived experience on the findings being generated.

First, all interview and focus group data were transcribed and reread to allow the first author to achieve familiarization with the data, while also adding descriptive annotations of what participants were saying to inform the second stage. Second, data coding was undertaken by collating excerpts of data from all interviews and focus groups. Third, once all data had been coded, codes were considered together and organized into preliminary themes that captured patterns of their content and meaning. Fourth, the preliminary themes were refined by iteratively comparing them against the generated codes and entire dataset. Fifth, after the themes were refined, the lead researcher engaged in a deductive process to aid interpretation and labeling of the theme contents. Specifically, sub-themes were reiteratively compared with one another and pre-existing theory, as this allowed for amendment and reinterpretation into overarching analytical themes that were distinct and relevant to the research questions. Sixth, quotes that were deemed to be representative of the labeled themes were identified and used to support data interpretation with reference to the research questions.

## Results

The results represent the collated responses from all interviews and focus groups. Findings from the seven sub-themes generated during thematic analysis are grouped under two higher-order analytical themes to provide an insight into how social support and social identities influence adaptations to stress: (1) social factors influencing the nature of social support, and (2) social factors influencing the provision and receipt of social support. Quotes typical of each theme using pseudonyms are also presented to illustrate interpretation and synthesis.

### Analytical theme 1: Social factors influencing the nature of social support

#### Sub-theme 1.1: Influence of identity

Co-ordinators and teams of support staff explained how the Academy was situated within an organization (NGB) that had a remit of supporting performance alongside the welfare of its players, and that this remit defined who they were (their identity); *“Going back to our responsibility as a governing body, that we have to develop better people as well as better players”* (John, Co-ordinator). Co-ordinators, teams of support staff, and players indicated that this remit and identity also defined what stressors and support needs were relevant and salient to them; *“Sometimes that can be personal things, sometimes it can be lifestyle-type situations, sometimes it can just be ill-informed choices, you’re dealing with young inexperienced people who have their sights very much on that starry vision of being a professional player”* (Jane, Co-ordinator).

The Academy’s resulting social identities seemed to, in turn, influence the form and provision of social support such that it had perceived benefits for their support needs (i.e., resulted in beneficial performance and player welfare outcomes). For example, co-ordinators and support staff indicated how social support within the Academy was multidisciplinary in nature and designed to have a complimentary focus on rugby, dual career, and personal development; *“when we do quarterly reviews, we’re looking at things like work-ethic or responsibility* […] *that’s obviously not just rugby its overall making them, we’re looking at them being independent. Or, you know, good human beings who are gonna get jobs in other roles”* (Richard, Physiotherapist). Players also reported receiving support that had *“a 50-50 balance, like 50% is focused on training. But, the other 50% is focussing off-the-pitch as well”* (Rickie, Player). For example, one player described how“[my Academy Manager]’s *been driving me quite hard for my studies and making sure that they’re on track and they’re going as well as they can* […] *it’s very focused on, your studies come first and you train around that, rather than you train then your studies come around your training.*” (Nemo, Player).

#### Sub-theme 1.2: Influence of context

Although there was a consistent national identity and social support structure throughout the NGB, there were also regional variations in terms of the stressors and available resources faced by different sub-groups (e.g., rural vs. metropolitan Academy training centers). For example, one Academy Manager (Adam) said *“rather than just being an Academy that is ‘part of* [NGB] *Rugby’, which is where it started with, now we’re trying to develop our own identity, and then something that actually sits back, about ‘yes we’re not any different to what’s going on, but we are different’* […] *people have the perception that the* [region] *players aren’t fit, so we want them to be athletic, but we want to be mean, and then we’ve got that word ‘dogged’ that comes from it, and we think that encompasses us a region.”*

In other words, while there appeared to be a general alignment with a super-ordinate NGB identity, co-ordinators and support staff indicated that the operationalization of this super-ordinate identity was regionally distinct; *“We have to have a national programme that’s delivered regionally* […] *the academy structure that we have is very much a national focus, national messages, delivered regionally, that does reflect the region that the players are in”* (Robert, Co-ordinator). These regionally distinct identities were often manifested and characterized through the use of “Academy Values”, which were used to create regional variations in the support behaviors of staff and players. For example, one Academy Manager from a rural training center (Marlin) spoke about their Academy Values as being “Developing and Sharing Knowledge” because they needed to utilize a bigger network of support providers (“*‘Developing and Sharing Knowledge’ is really important because we couldn’t run our Academy with just our staff”*), and “Combining Professionalism” because they needed to support “*transitioning players in*[to] *a professional environment, we want to make sure they understand what professionalism is.”* As such, while the provision of social support aligned with the super-ordinate NGB identity, there were also regional variations in the provision of support between each Academy. These regional variations allowed each Academy to address the stressors and support needs that were most pertinent to them.

#### Sub-theme 1.3: Influence of support network

Support networks were structured to achieve beneficial outcomes that were consistent with each Academy’s identity and support needs. For example, support staff emphasized the importance of cultivating close relationships with other staff to support holistic player development (i.e., a beneficial, identity-consistent outcome). Similarly, support staff would curate relationships with external support providers to (for example) assist with players’ educational attainment;“[I] *asked Marlin like what I could do* [about educational attainment], *how I could juggle it, and then I’ve just had a meeting with* [support staff] *about potential, potential ideas and they’re gonna put me in touch with the guy in* [city] […] *he has a good education background so they said he’ll help a lot”* (Sean, Player). Conversely, support staff indicated that high levels of parental support may prevent players from developing autonomy skills; “*now, you’re far more likely to have the parent phoning you directly saying ‘how does this happen, how does that happen?’ and their far more inquisitive which means the actual young person’s actually got a lesser skillset of that*” (Jordan, Academy Manager). Support staff would, therefore, encourage players to reduce their level of reliance on parents in order to support players’ holistic development through becoming more autonomous and self-reliant; *“I think the way we do that generally is we put a little bit of onus back on the player* […] *to take some ownership of that so we’re not chasing them every week”* (Pete, Academy Manager).

### Analytical theme 2: Social factors influencing the provision and receipt of social support

#### Sub-theme 2.1: Provision and receipt of support in groups

Both co-ordinators and support staff worked closely together to achieve a multidisciplinary understanding of—and provision for—players’ support needs. As such, the provision and receipt of social support in the Academy operated as a dynamic exchange within groups. For example, support staff described how *“We pretty much talk through all our players, needs are highlighted and discussed, so if we’ve got a concern about a player, whether it’s a sport-specific concern or a behavioural concern, or something in their life out-with this, we can monitor that either formally or informally”* (Jordan, Academy Manager).

Support staff and players indicated that support exchanges within groups often resulted in positive adaptations to stress due to being *responsive*. This was because the group context allowed for salient stressors to be easily identified. A physiotherapist (Richard) shared an example of this; *“‘This player feels low’, the physio said this, and the coach then said ‘how’d ya know?’, and I was like, ‘well, it’s my perception, I’ve grown to know a person coming in day-to-day and the last two or three weeks I’ve noticed there’s been a change’.”* Similarly, group-based support was considered to be *dynamic*, as it enabled appropriate providers or types of support (e.g., vocational support) to be easily prioritized depending on the most salient stressor (e.g., if a player became long-term injured). For example, one coach (Magnus) imitated what this dynamic group-based support provision looked like; *“‘we need to obviously intervene here’. Who is it?* [Coach]*’s got the better relationship with him, you’ll maybe pick that up, or in situ*, [Physiotherapist]*’s just picked it up as they flag.”* Players also indicated that support exchanges within groups often resulted in positive adaptations to stress due to being *accessible*. Specifically, the group environment allowed for members to easily access and engage with a range of support providers or recipients (e.g., *“coaches and physios are people I’d see pretty much every day when I’m training, so, if anything goes wrong, they’re always there”*; Hugh, Player).

#### Sub-theme 2.2: Provision of normative social support

The Academy identities and their characterizing identity content (e.g., Academy Values) influenced what behaviors were considered normative and meaningful amongst groups of support staff and players. These group norms in turn influenced the provision of normative social support from staff; “[If players’] *behaviours and it’s not quite meeting what we’re expecting, I have the ability that – we as a team, we talk a fair amount and class our Values and what we’re aiming for – and I can project that through to the athlete when we’re on the physio plinth, so to speak, and go: ‘well, do you think that’s a wise choice?”’* (Brian, Physiotherapist).

The meaning of normative social support provision to the group’s identity meant that staff would provide additional support over and above their remit if doing so aligned with their group’s “Academy Values.” For example, if a player’s behavior (e.g., displaying autonomy and good work ethic) aligned with their Academy’s values (e.g., “accountability”), this would be rewarded by warranting additional support from staff; *“we’ll give them a little bit more additional S&C support, we will give them a bit of nutritional advice, they will potentially see* [physiotherapist] *and get a bit more support which they’re not ‘technically’ in terms of that level due to get, and I think if we were to be honest about it, I think the Values does shape the support we give them”* (Adam, Academy Manager). The converse was also noted, where less support would be provided if behaviors did not align with the group’s Values; *“Certainly, from my point of view that Identity has a big impact on what I deliver to them. For example, there’s a* [player] *guy – they’re given supplements – that* [player] *guy I was giving the supplements from, he doesn’t get them because the other parts, ‘turn up on time, bring the effort, bring the right kit’, those three parts weren’t being delivered by him*” (William, S&C Coach).

#### Sub-theme 2.3: Receipt of normative support

Co-ordinators, support staff, and players indicated that the groups which players belonged to—and their characterizing identities—influenced individual players’ receipt of social support. For example, co-ordinators and support staff indicated that players’ understanding of support were often based on the vicarious experiences of their teammates (e.g., *“in terms of actual* [mental health] *support and knowing what’s available, I don’t think I know much about it, just generally, because it’s never happened to any of my friends or any sort of thing”*; Oliver, Player). Further, their engagement with different types of support were also influenced by the extent to which doing so aligned with group norms (e.g., Academy Values); “[The coaches are] *working hard to make sure we’ve got everything that we need and we kind of repay them by following these Values and sticking to what we’re told.”* (Sheila, Player).

This had implications for how social support influenced resultant adaptations to stress. Notably, players showed a better understanding of and engagement with normative social support (e.g., rugby-related support such as coaching), which would facilitate positive adaptations to rugby-related stressors. Conversely, this meant that players had a poorer understanding of and engagement with non-normative social support (e.g., mental health or performance lifestyle support), thereby posing a greater risk for negative adaptations to non-rugby stressors; *“they want to be rugby players, they don’t necessarily get the rehab and prehab and it’s, they’re all at different stages and most of the younger players that we deal with are, are just ‘rugby, gym’ and that’s all they see as important”* (Derek, Coach).

Support staff described how engaging with non-normative support could result in players experiencing negative adaptations to stress due to concerns over how it may be perceived or affect their standing within their group (e.g., being perceived as ‘weak’ and risking deselection); “[There is] *a barrier there depending on the type of support you’re referring for, whether players see it as being related to performance. You know, we’ve had a number of conversations with the players that blows up* [imitating a conversation between coach and player]*: ‘why don’t you come and speak to us?!’, ‘oh, well, oh. I didn’t want to, didn’t want to discuss that with you!”’* (Pete, Academy Manager). These concerns about engaging with non-normative support were also corroborated by some players; *“that’s the big problem at the moment, I would say, mental health stuff probably is available, but you have to go and seek it yourself, and people might not feel too comfortable doing that”* (Irene, Player).

#### Sub-theme 2.4: Support exchanges between ingroup members

Co-ordinators, support staff, and players indicated that social support exchanges resulted in more positive adaptations to stress when support provider and recipient identified with one another on some valued dimension (e.g., playing experience, credibility, etc.). First, this seemed to be because shared identification helped to create rapport and trusting relationships between provider and recipient. For example, one player (Ricky) explained how having a sense of shared identity made it easier to overcome help-seeking stress; *“It makes you more comfortable in asking questions and stuff like that, so I’d feel really comfortable talking to one of these coaches about, just going up to them and asking ‘right, I’m doing, I’m struggling with this’* […] [Interviewer: *“Why does that make you comfortable?”*] *It’s just, it’s easy. It, as I say like, support is making things easier, it’s just easy and there’s no stress”.* This, in turn, allowed for the stressors and support needs of players to be better understood by support staff, thus enabling positive adaptations to stress.

Second, the extent to which support providers were considered an ingroup member (“*one of us*”) influenced the perceived trustworthiness and credibility of support. Co-ordinators, staff, and players indicated that (particularly non-normative) support was more graciously received and engaged with when provided by team-mates or retired professional players due to their level of shared knowledge and experience (e.g., of playing rugby and/or working directly with athletes); *“somebody from HR* [human resources] *trying to sell this* [non-rugby support] *to players is not going to work* […] *they’re all sitting there half asleep. You put in a couple of ex-rugby players up there saying it to them? They’re listening.*” (Michael, Co-ordinator). This had implications for the perceived effectiveness of support provided; *“one of the pro team coaches which a lot of these guys will really look up to might have a message, and they might instantly buy into that”* (Jordan, Academy Manager). For example, one player (Jerry) explained why he trusts the support received from his S&C coach (a former international rugby player); *“I respect* [S&C coach] *massively because he’s top class 7’s player, he knows what he’s talking about, and I feel yeah, cause it obviously, I do trust what he says because he’s been there and done that”.*

## Discussion

The purpose of this study was to explore how social support and social identities influence adaptations to stress in a Rugby Academy Programme. This study has contributed to the extant literature by demonstrating that group-based perceptions of social support influence adaptations to stress. Specifically, whether social support influences positive, negative, or indifferent adaptations to stress depended on (1) social factors influencing the nature of social support, and (2) social factors influencing the provision and receipt of social support. In the following sections, we build a sequential discussion of these findings using the study’s three research questions.

### RQ1: How can we understand social support using the social identity approach?

A better understanding of how social support influences adaptations to stress can be gained by appreciating that social support is a dynamic exchange between providers *and* recipients, and may often occur in group settings (for example, between multidisciplinary support teams and athletes; sub-theme 2.1; Reid et al., 2004). This means that both the nature (e.g., structure and function) of social support, as well as the provision and receipt of social support, will be influenced by social factors (i.e., group identity-processes). In this regard, our findings indicate that the contexts which groups find themselves in influence what stressors and support needs they consider to be most pertinent (e.g., sub-theme 1.2), and that their group identities influence what support they consider to be most beneficial for addressing those stressors and support needs (e.g., sub-theme 1.1). Further, our findings emphasize that social support can be understood in social terms by considering whether the provision and receipt of social support is deemed normative/non-normative, and whether there are shared social identities between providers and recipients. While most of the extant literature has documented the experiences of social support recipients, our attempt to capture both perspectives of providers (enacted support) and recipients (perceived and received support) within groups has highlighted how these social factors influence the effects of social support (this is discussed subsequently).

### RQ2: How can social factors influence the effects of social support?

Building on the evidence presented above, a better understanding of how social support influences adaptations to stress can be gained by considering how *social factors* (such as shared group memberships) influence the effects of social support. According to self-categorisation theory, group membership will motivate individuals to depersonalize and self-stereotype themselves in group-like terms (i.e., as an interchangeable group member; Turner, 1982; Turner et al., 1994). Depersonalization and self-stereotyping in turn provides the basis for mutual influence where group members will be motivated to achieve consensus over their social support behaviors and to mobilize those behaviors accordingly (e.g., “*this is how ‘We’ do support*”; Haslam et al., 1998). In relation to our findings, these theoretical tenets help to explain (first) how social factors influence what stressors and support needs are considered most pertinent to the group, and (second) how identity content influences the effects of social support.

First, our findings demonstrate how the stressors considered most pertinent to each regional training center (e.g., concerns over being unfit, not showing up to training on time, etc.) were incorporated into the superordinate NGB and Academy group identities (e.g., espousing values around “being planned and organized” and “professionalism and accountability”, respectively). In this way, group identities may allow group members to achieve consensus over what stressors are considered most pertinent to them (e.g., sub-theme 1.2; the need to support player welfare alongside performance), as well as what support is considered beneficial for abating those stressors (e.g., sub-themes 2.1 and 2.2; providing vocational support when a player is injured to achieve positive adaptations to welfare stressors). Social support’s influence on adaptations to stress may therefore depend on the degree to which it aligns with the group consensus regarding their most pertinent stressors (i.e., “*is this support beneficial to ‘Our’ needs or not?”*).

Second, our findings demonstrate that the effects of social support are influenced by the identity content of groups. For example, groups of players espousing values around being “self-reliant” and “autonomous” may constrain the structure of their support network (e.g., sub-theme 1.3, resulting in the modification of players’ engagement with certain support providers; sub-theme 2.4, players favoring engagement with support providers whom they have shared identities with). This might be because structural ties within groups are likely to be the basis for both beneficial *and* debilitative social support. Indeed, researchers have demonstrated that some social support ties might facilitate positive adaptations to stress, whereas others might facilitate negative adaptations to stress (Tucker et al., 2016; Arnold et al., 2018; Sarkar and Hilton, 2020). Therefore, identity content may influence the *structure* of social support by removing potentially deleterious sources (and thereby types) of social support, so that the support is more optimally matched to the ingroup’s perceived needs (Cutrona and Russell, 1990). Similarly, identity content may constrain the *function* of social support (e.g., NGB and Academy identities influenced what support was provided by co-ordinators and support staff, and how it was received by players; sub-themes 2.2 and 2.3). As above, this may be due to shared group membership/s resulting in the consensus and mobilization of normative social support behaviors. Social support’s influence on adaptations to stress may therefore also depend on the identity content of groups—notably because this social factor has implications for the structure and function of social support (Hartley et al., 2020).

### RQ3. How can we explain social support’s positive, negative, and indifferent adaptations to stress in sport?

The above helps to explain social support’s positive, negative, and indifferent adaptations to stress in sport. As discussed, our findings indicate that the effects of social support are influenced—positively, negatively, and indifferently—by the degree to which providers and recipients achieve consensus over the normative provision and receipt of social support and the implications thereof (Haslam et al., 1998). This is supported by social and health psychology literature demonstrating how group membership/s can facilitate social support and result in positive outcomes (e.g., research on the *social cure*; Haslam et al., 2018). Conversely, this is also supported by literature demonstrating how group membership/s can exacerbate social support and result in negative outcomes (e.g., research on the *social curse*; Kellezi and Reicher, 2011). Our findings demonstrate how, for example, providers gave more or less support depending on individual members’ compliance with group values (sub-theme 2.2), and that players were less likely to engage with mental health support due to concerns over how it would be perceived (sub-theme 2.3). As such, although well-intentioned and optimally matched social support *could* be available and provided (e.g., Cutrona and Russell, 1990), the identity-based implications behind the provision and receipt of particular types of support might determine whether it results in positive, negative, or indifferent adaptations to stress (Hartley et al., 2020).

Considering the above, achieving group consensus over normative social support behaviors may actually create *barriers* to engaging with non-normative forms of support and support providers. This explains why some types of support (e.g., mental health services) or providers of support (e.g., non-rugby providers) are not always sought or well-received in sport (sub-themes 2.3 and 2.4, respectively). This is because doing so may conflict with the group’s identity-based norms (e.g., of being a high-performing player), and attempting to access non-normative support (e.g., mental health services) may result in negative adaptations to stress (sub-themes 2.2 and 2.4; Wood et al., 2017). Literature on identity-based threat suggests this may be because identity counter-firming support is likely to be considered *threatening* to the ingroup and evoke group disapproval (e.g., accessing mental health services might conflict with the identity of being a high-performing player and thus create concerns over deselection; Butler et al., 2018). This highlights that, although social identities are often considered beneficial for mental health and wellbeing (e.g., Haslam et al., 2018), they might also lead to negative outcomes because individuals don’t want to go against their group’s norms (e.g., a *social curse* effect; Kellezi and Reicher, 2011). Therefore, considering the identity-based implications behind the provision and receipt of some forms of social support (e.g., whether it is normative, non-normative) may allow us to explain social support’s positive, negative, and indifferent adaptations to stress in sport.

Similarly, this explains why the provision of social support from certain *providers* (and not others) may achieve positive, negative, or indifferent adaptations to stress. Notably, certain support providers (e.g., mental health specialists) might be deemed non-normative due to being incongruent with a group’s identity content (e.g., values centered on performance), thereby reducing the likelihood that their support would achieve positive adaptations to stress (or indeed whether group members engage with them in the first place). Equally, when there is a shared social identity between support provider and recipient, the identity-based implications behind such an exchange might allow for rapport, trust, and credibility to emerge more favorably (i.e., due to interacting with an ingroup member, sub-theme 2.4; Foddy et al., 2009; Greenaway et al., 2015). Extant literature might suggest that social support results in *positive* adaptations to stress when there is common ground of shared social identity between a provider and recipient (e.g., Haslam et al., 2005; Nicholson et al., 2011). However, our findings support the notion that social support’s resultant adaptations to stress will simply be more *potent* when there is a shared social identity. This is because although an ingroup provider might be deemed to be more trustworthy and credible, they may not have sufficient knowledge and expertise to provide effective support. Indeed, an ingroup provider might provide unintentionally *harmful* support that results in negative adaptations to stress due to its perceived trustworthiness and credibility (e.g., sub-theme 2.4; a professional rugby player telling an aspiring player to focus exclusively on rugby at the expense of developing their dual career).

### Strengths and limitations

This study had several strengths. First, the use of qualitative methods allowed for a unique contribution to extant literature by allowing us to capture social identity “in action.” Indeed, the naturalistic study design allowed the researcher to build rapport with participants and recognize the subtleties of what was being said, thereby offering rich and contextualized insights into how social support and social identities influence adaptations to stress (Rubin and Rubin, 2011). Second, capturing both perspectives of providers (enacted support) and recipients (perceived, received support) helped us to understand how social factors influence the effects of social support. This, in conjunction with the social identity approach, helped to address the limitations of extant approaches to the study of social support and adaptations to stress in sport. Third, the use of purposive sampling allowed the views of understudied individuals (e.g., co-ordinators and support providers) with distinct yet important roles in the provision and receipt of social support to be captured.

With regards to limitations, due to the study’s naturalistic design there were no observational data to supplement participants’ accounts of providing and receiving social support. However, it is worth noting that studies which have combined observational and self-report measures of social identity-related processes have reported high correlations between these indices (e.g., Reicher and Haslam, 2006). Second, although this study sampled almost the entire Academy co-ordinator and support staff sub-populations, it nonetheless contained a convenience, self-selected sample of Academy players which may have increased the risk of sampling bias. Third, although steps were taken to strengthen rigor and impartiality during the phases of data collection and analyses (e.g., using co-authors as critical friends; Tong et al., 2007; Smith and McGannon, 2018), there remains the possibility that the researchers influenced the insights generated (e.g., our own biased experiences of social support provision and receipt may have influenced data interpretation).

### Future research

While our findings offer naturalistic and rich insights into how social support and social identities influence adaptations to stress, it will be necessary for researchers to replicate and further investigate these findings using other methods and paradigms of research enquiry. In this regard, it may be important to consider the following. First, much of the extant literature adopts a singular perspective of social support (perceived or received support). As such, a fuller understanding of how the provision and receipt of social support influences adaptations to stress (and other outcomes) could be gained by simultaneously investigating enacted, perceived, and received support. Second, it is likely that relational differences exist with regards to the social support experience (i.e., due to perceived disparities between the assistance given by support providers and the impact it has on support recipients). To get a more accurate understanding of the social support experience, researchers could account for these relational differences by capturing the perspectives of important yet understudied individuals (e.g., the views of support providers, support co-ordinators, or other stakeholders such as teammates or parents). While qualitative methods might allow for naturalistic insights into these perspectives, it may also be possible to capture them by comparing singular measures of social support (e.g., Freeman et al., 2014) with team-referent measures (e.g., Coffee et al., 2017). Third, our findings reinforce existing evidence for how social factors (such as social identities) influence social support’s effects. A better understanding of social support’s effects could therefore be gained by investigating how it is influenced by other factors (e.g., systemic or political constraints on available resources, the physical layout of support environments, or the influence of hierarchy on support exchanges, etc.).

## Implications and conclusion

By drawing on the social identity approach, the present study makes a novel contribution toward better understanding how social support and social identities influence adaptations to stress in a Rugby Academy Programme. Specifically, the present study explains how adaptations to stress in sport are influenced by the identity-based implications behind the provision and receipt of social support. This has several implications for how organizations, teams, and practitioners can support positive adaptations to stress in sport. First, our findings demonstrate that the experience of social support is embedded in the social nature of group life. It is thus important to consider the influence of group identity-processes on social support’s effects. Second, attempting to understand the context and identities of particular groups (e.g., their characterizing identity content as operationalized through “team values” or other artifacts), may aid identification of a group’s most pertinent stressors along with the types of normative support that may result in positive adaptations to stress. Equally, doing so may aid identification of the types and providers of support that are deemed *non-*normative (e.g., mental health support), and thus less likely to be engaged with by group members. Third, social support may be more *potent* when there is common ground of shared social identity (e.g., an athlete receiving coaching advice from a former-athlete) due to the identity-based implications behind the provision and receipt of social support—which poses both opportunities and challenges to achieving positive adaptations to stress. More concretely, our findings suggest that sport organizations, teams, and practitioners could enhance the effectiveness of social support by championing and embedding multifaceted group identities centered on desired outcomes (e.g., improving *both* performance and wellbeing). Doing so may allow for more types and providers of support (e.g., psychological, medical, dual career advisors, etc.) to be considered normative “parts of the ingroup,” and thereby more readily and effectively engaged with by athletes. In sum, group-based perceptions of social support influence adaptations to stress.

## Data availability statement

The raw data supporting the conclusions of this article will be made available by the authors, without undue reservation.

## Ethics statement

This study, involving human participants, was reviewed and approved by the General University Ethics Panel at the University of Stirling. Written informed consent to participate in this study was obtained from all participants (and participants legal guardian/next of kin where relevant). Written informed consent was obtained from the individual(s) for the publication of any potentially identifiable data included in this article.

## Author contributions

This research comprised part of CH’s doctoral thesis. As such, CH led the design, data collection, analysis, and write up of the research. PC and PA first and second supervisors, respectively, provided guidance and supervision on the research. This research was undertaken while PC was previously employed with the Faculty of Health Sciences and Sport at the University of Stirling. All authors contributed to the article and approved the submitted version.
